# Existential threat and responses to emotional displays of ingroup and outgroup members

**DOI:** 10.1177/13684302221128229

**Published:** 2022-11-05

**Authors:** Janet Wessler, Job van der Schalk, Jochim Hansen, Johannes Klackl, Eva Jonas, Maurice Fons, Bertjan Doosje, Agneta Fischer

**Affiliations:** 1 German Research Center for Artificial Intelligence, Germany; 2 Cardiff University, UK; 3 University of Salzburg, Austria; 4 University of Amsterdam, the Netherlands

**Keywords:** affiliation, emotional displays, facial behavior, mortality salience, outgroup derogation

## Abstract

The present research investigates how emotional displays shape reactions to ingroup and outgroup members when people are reminded of death. We hypothesized that under mortality salience, emotions that signal social distance promote worldview defense (i.e., increased ingroup favoritism and outgroup derogation), whereas emotions that signal affiliation promote affiliation need (i.e., reduced ingroup favoritism and outgroup derogation). In three studies, participants viewed emotional displays of ingroup and/or outgroup members after a mortality salience or control manipulation. Results revealed that under mortality salience, anger increased ingroup favoritism and outgroup derogation (Study 1), enhanced perceived overlap with the ingroup (Study 3), and increased positive facial behavior to ingroup displays—measured via the Facial Action Coding System (Studies 1 and 2) and electromyography of the zygomaticus major muscle (Study 3). In contrast, happiness decreased ingroup favoritism and outgroup derogation (Study 2), and increased positive facial behavior towards outgroup members (Study 3). The findings suggest that, in times of threat, emotional displays can determine whether people move away from unfamiliar others or try to form as many friendly relations as possible.

When confronted with existential threat, individuals typically distance themselves from others who are outside their social circle (e.g., Greenberg et al., 1997). At the same time, when facing threatening situations, people search for others’ help, security, and comfort (e.g., Schachter, 1959; Wisman & Koole, 2003). The emotions of others might become particularly relevant in such situations because they can serve as cues for where the danger is coming from (Keltner & Haidt, 1999) and may also give insight into others’ (non)affiliative intentions (Fischer & Manstead, 2008). Yet, previous research has rarely examined the role of the emotional context in determining the reactions towards threat. The current research investigates how emotional displays of ingroup and outgroup members regulate distancing from or affiliation with these groups when people are confronted with existential threat.

## Responses to Mortality: Worldview Defense and Affiliation

Human beings, like all animals, have an instinctive drive for self-preservation. At the same time—and probably different from other animals—they know that they are eventually going to die. According to terror management theory (Greenberg et al., 1986, 1997; Solomon et al., 1991), the conflict between the human drive to survive and humans’ awareness of their mortality produces the potential for overwhelming anxiety (i.e., terror). In order to manage this anxiety, the theory proposes two anxiety-buffering systems: (a) cultures that offer worldviews and standards of value that can provide meaning beyond an individual’s existence, and (b) individuals’ self-esteem, which is defined as the extent to which one lives up to one’s cultural value system. The theory assumes that increased accessibility of death-related thoughts will result in a stronger need to affirm one’s cultural worldviews and to bolster one’s self-esteem. This traditional view of terror management theory has been tested extensively (for a meta-analysis, see Burke et al., 2010). After inducing mortality salience (e.g., via two open-ended questions about one’s death) and a delay task, participants typically defend their moral or cultural values. For example, they show increased ingroup favoritism (Greenberg et al., 1990), demand stronger punishments for people who violate their cultural norms (Florian & Mikulincer, 1997; Rosenblatt et al., 1989), and exhibit more aggression towards individuals who criticize their cultural beliefs (McGregor et al., 1998). Similarly, in order to bolster a positive self-image, they prefer to associate themselves with groups that provide a feeling of self-worth (e.g., Taubman Ben-Ari et al., 1999), while distancing themselves from groups that influence their self-worth negatively (Dechesne et al., 2000). Thus, generally speaking, mortality salience increases ingroup favoritism as well as outgroup derogation.

Apart from these “classic” findings on the detrimental effects of mortality salience on perception and behavior towards outgroup members, research on terror management has also shown that under certain conditions, mortality salience can increase affiliation with others (for an overview, see Vail et al., 2012). For example, after mortality salience, people were more willing to initiate social interactions (Taubman Ben-Ari et al., 2002) and reported higher romantic commitment to their partners (Florian et al., 2002). The enhanced affiliation need after mortality salience even leads people to accept worldview-opposing individuals as a source of affiliation (Wisman & Koole, 2003).

Past research has proposed two important contextual factors that can moderate whether people react with worldview defense or with affiliation strategies under mortality salience. First, when norms or values promoting positive intergroup attitudes are chronically or situationally accessible, mortality salience can bolster such outcomes (Jonas et al., 2008). For example, mortality salience reduced derogation of a U.S. critic when U.S. individuals had earlier completed a questionnaire on tolerance (Greenberg et al., 1992), and led to more favorable attitudes amongst White participants towards Black individuals when egalitarianism had been primed (Gailliot et al., 2008). Second, establishing personal connections or making a superordinate group identity salient can initiate a recategorization mechanism which leads people to expand their group boundaries and include members of otherwise different groups into their ingroup (Gaertner & Dovidio, 2000; Gaertner et al., 1993). After mortality salience, making a common superordinate group identity salient can decrease outgroup derogation (Giannakakis & Fritsche, 2011; Motyl et al., 2011).

In sum, previous findings on the effects of mortality salience on distancing from and affiliation with outgroup members point in two different directions: When reminded of their mortality, people show either worldview defense strategies (i.e., derogation of an outgroup) or, under certain conditions, a tendency to affiliate with and include others (even worldview-threatening group members). We propose that others’ emotional displays might be an additional contextual factor that determines whether people respond with distancing or affiliation after mortality salience. In times of threat, subtle emotional signals might help to decide whether to exclude potential enemies or form as many friendships as possible.

## Social Functions of Emotions: Distancing and Affiliation

From a functional perspective, emotional expressions are important for effective social interaction. Emotional displays are meaningful social signals that inform the perceiver about the expresser’s appraisal of the situation and their behavioral intentions, thus influencing the perceiver’s emotions and behavior (e.g., Keltner & Haidt, 1999; Manstead & Fischer, 2001; Schwartz & Clore, 1983; van Kleef, 2009). Emotions can fulfill two basic functions in social contexts (Fischer & Manstead, 2008): (a) protecting and enhancing one’s social standing via distancing from others, or (b) forming and maintaining social relationships via affiliation with others. For example, emotions such as anger and contempt can be expressed to signal dominance, whereas expressions of happiness and sadness signal the expresser’s willingness to affiliate (Fischer & Manstead, 2008; Fridlund, 1994; Knutson, 1996). On an intergroup level, comparably, emotions can define group boundaries (Keltner & Haidt, 1999): Anger displays, for example, can sharpen group boundaries, while happiness displays can override them (e.g., Bourgeois & Hess, 2008). For example, individuals tend to automatically imitate anger displays of ingroup members more than those of outgroup members, while they automatically imitate happiness displays of ingroup and outgroup members to a similar degree (e.g., Bourgeois & Hess, 2008; van der Schalk, Fischer, et al., 2011). It has similarly been suggested that evolutionary processes have selected angry faces, as signals of threat, to be processed highly rapidly and efficiently in order to prevent escalation of conflict in social groups (Öhman, 2009).

## Hypotheses and Overview of the Present Research

Combining insights and empirical findings from terror management theory and the social functions of emotions, we propose that emotional signals regulate how individuals respond to others under mortality salience. We hypothesize that mortality salience will elicit worldview defense when individuals are exposed to anger displays. In particular, we predict that both ingroup favoritism and outgroup derogation will increase. In contrast, we hypothesize that mortality salience will instill a general affiliation tendency when individuals are exposed to happiness displays. As such, we predict that ingroup favoritism and outgroup derogation will decrease.

We also investigated how the effect of mortality salience and emotion displays on worldview defense and affiliation need would be influenced by the social category of the expresser. We predicted that the effect of mortality salience on worldview defense (increased ingroup favoritism and outgroup derogation) would be more pronounced after outgroup anger displays than after ingroup anger displays. For the effect of mortality salience on affiliation need (i.e., attenuated ingroup favoritism and outgroup derogation), we had no specific predictions regarding the moderating effect of ingroup versus outgroup happiness displays.

We conducted a series of studies to test these hypotheses. In Studies 1 and 2, we presented participants with a mortality salience or control (dental pain) manipulation before exposing them to anger (Study 1) or happiness (Study 2) displays. These emotions were displayed by either ingroup or outgroup members. Ingroup favoritism and outgroup derogation were measured with self-report measures and facial behavior with the Facial Action Coding System (FACS; Ekman & Friesen, 1978). In Study 3, we made some methodological changes and added a physiological measurement of participant’s facial reactions (i.e., electromyography).

## Study 1

### Method

#### Disclosure statement

The following sections provide a brief overview of the materials and procedure. For a complete overview of the methodology, see Supplemental Material S1.

#### Participants and design

The study had a 2 (mortality salience: experimental vs. control) x 2 (social category: ingroup vs. outgroup) between-subjects design. For reasons of convenience, we planned to collect data of 25 participants per group. There were 101 participants (77 female; *M*_age_ = 21.83, *SD*_age_ = 4.96). Participants were recruited at the University of Amsterdam and received either course credit or €3.50 (approximately US$5.00 at the time) for their participation. The final sample consisted of 94 participants after exclusions.^1^ A sensitivity analysis revealed that with a significance level of α = .05 and a power of (1 − β) = .80, this was sufficient for an effect size of *f* = .29.

#### Materials

##### Manipulations

As a manipulation of mortality salience, participants received two open-ended questions about what they thought would happen to them if they died, and the feelings they experienced when they thought of their own death (Greenberg et al., 1990). Participants had 3 minutes to answer each question. In the control condition, participants answered similar questions about a visit to the dentist.

As main task in the study, participants were told that they would view short film clips of four models, and they were asked to form an impression of these models. The instructions stated explicitly that the models were either Dutch (an ingroup for Dutch participants) or Moroccan (an outgroup for Dutch participants; Dotsch et al., 2008). Participants were then shown film clips of anger displays of four male models from the Amsterdam Dynamic Facial Expression Set (ADFES; van der Schalk, Hawk, et al., 2011). Depending on social category condition, participants viewed emotional displays of either White or Mediterranean models. The film clips were 6.0s in length and started from a neutral expression, with the onset of the emotion displays at 0.5s and reaching the apex at about 1.0s. The film clips were presented consecutively, with an intertrial interval of 1s.

As manipulation checks, participants indicated the extent to which the models seemed native Dutch or nonnative Dutch on a 7-point scale (1 = *not at all*, 7 = *very much*), and their perceived overlap with Dutch and Moroccan people using the Overlap of Self Ingroup and Outgroup Questionnaire (OSIO; Schubert & Otten, 2002) on a scale from 1 to 7. As a manipulation check of emotion, participants indicated the perceived intensity of anger (Study 1) and joy (Study 2) in the stimuli they had been exposed to, on a 7-point scale (1 = *not at all*, 7 = *very much*).

#### Measures

While watching the film clips, participants were unobtrusively filmed, and facial behavior was FACS coded (Ekman & Friesen, 1978). Frequency and intensity of AU4 (brow lowerer, associated with frowning), AU6 (cheek raiser and eye lid compressor, associated with smiling), and AU12 (lip corner puller, associated with smiling) were scored and combined to create facial activity scores.

To measure attitudes towards Dutch and Moroccans, participants indicated the extent to which they thought positively or negatively about these groups on a 7-point scale (1 = *very positive*, 7 = *very negative*).

At the time of the study, there was much public debate about a Dutch politician who is opposed to immigration from Muslim countries and in particular Morocco, and who was making a film about Islam in which he aimed to show that the Koran is a “violent and fascist book.” As a measure of outgroup derogation, we asked participants the extent to which they agreed with this opinion (1 = *not at all*, 7 = *completely*).

#### Procedure

The study was administered via a PC. Participants provided informed consent at the start of the study. After an initial questionnaire, participants were presented with the mortality salience manipulation, followed by a delay task: The Interpersonal Reactivity Index (Davis, 1980; see also Burke et al., 2010). They then answered a questionnaire measuring self-reported emotions and the main task of the study started: Participants viewed the emotional display film clips, indicated their self-reported emotions for a second time, and rated the White and Mediterranean models on several dimensions. Manipulation checks of social category condition and intergroup attitude measures were then administered. In the final part of the experiment, a manipulation check of perceived emotions was administered, and demographic information was collected. This included the measure of outgroup derogation. At the end of the study, participants were debriefed and received course credit or were paid.

### Results

#### Disclosure statement

Additional results are reported in Supplemental Material S2. There, we report findings of a “thermometer” measure of group favorability, liking of models, and self-reported emotions.^2^

#### Manipulation checks

Paired *t* tests revealed that Northern European models were perceived as more native Dutch (*M* = 5.65, *SD* = 0.87) than the Mediterranean models (*M* = 2.93, *SD* = 0.87), *t*(93) = 18.60, *p* < .001, η^2^ = .79, and that Dutch participants perceived more overlap with Dutch people (*M* = 5.00, *SD* = 1.14) than with Moroccans (*M* = 2.64, *SD* = 1.03), *t*(93) = 17.70, *p* < .001, η^2^ = .77, showing that the manipulation of social category was successful. Furthermore, a paired *t* test revealed that participants perceived more anger (*M* = 4.80, *SD* = 1.19) than happiness (*M* = 1.76, *SD* = 0.81) in the emotion displays, *t*(93) = 17.98, *p* < .001, η^2^ = .78, demonstrating that the emotion displays were identified correctly.

#### Attitudes

A 2 (mortality salience, between-subjects) x 2 (social category, between-subjects) x 2 (attitude target: Dutch vs. Moroccan, within-subjects) mixed ANOVA on the measure of positive and negative thoughts revealed a significant main effect of attitude target, *F*(1, 90) = 29.93, *p* < .001, η^2^ = .25. Overall, Dutch participants thought more positively about Dutch people (*M* = 3.43, *SD* = 1.16) than about Moroccans (*M* = 4.13, *SD* = 0.99).

**Figure 1.**
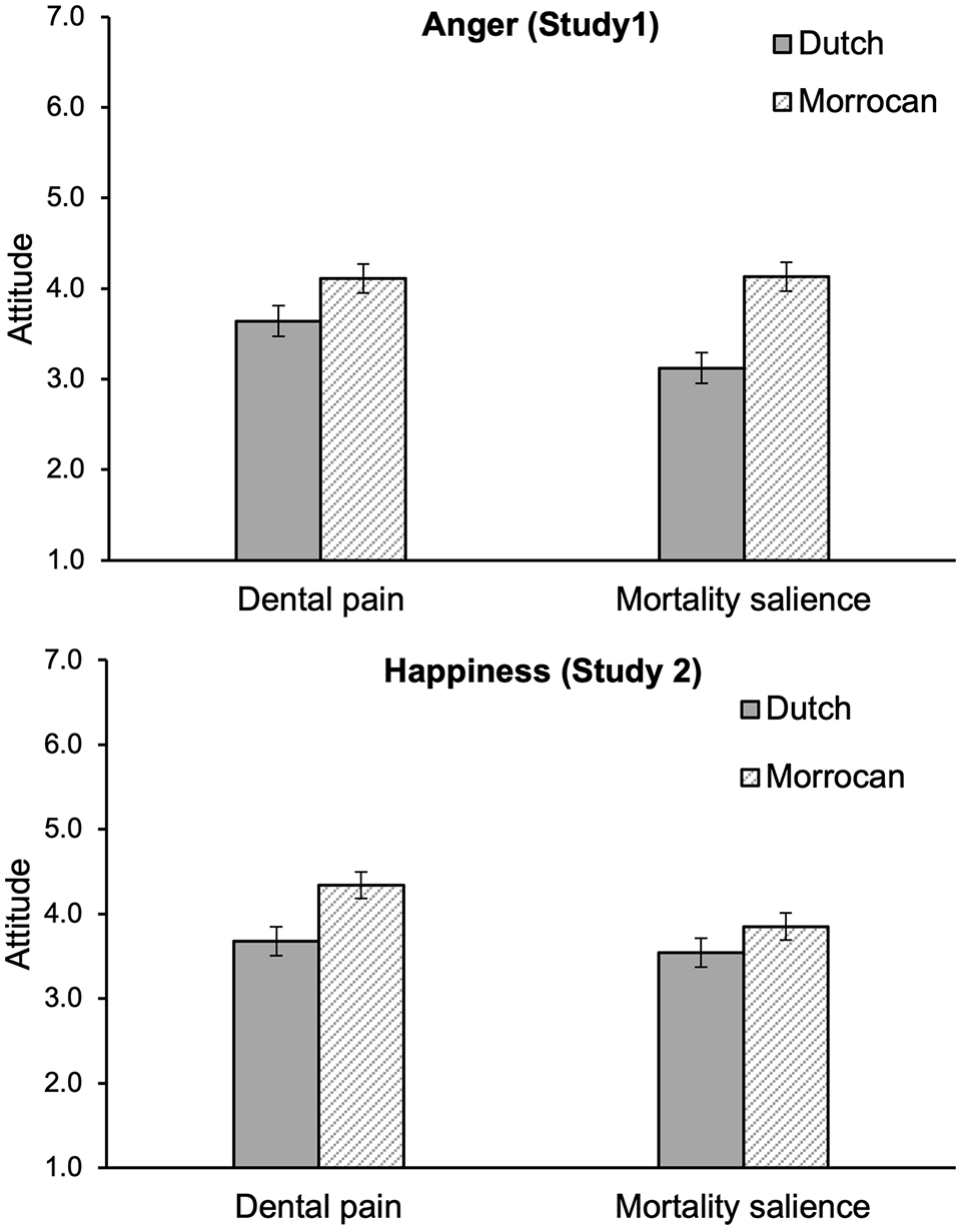
Attitude towards Dutch and Moroccans as a function of mortality salience for Study 1 (anger displays, top panel) and Study 2 (happiness displays, bottom panel). *Note*. Error bars represent ±1 *SE*.

The main effect of attitude target was qualified by a marginal significant two-way interaction between mortality salience and attitude target, *F*(1, 90) = 3.37, *p* = .070, η^2^ = .04. Figure 1 displays the combined effect of attitude target and mortality salience (top panel). Follow-up simple effects analyses revealed that, in the control condition, attitudes towards Dutch people were more positive (*M* = 3.66, *SD* = 1.24) than attitudes towards Moroccans (*M* = 4.13, *SD* = 1.06), *F*(1, 90) = 6.60, *p* = .012, η^2^ = .07, and that this effect was more pronounced in the mortality salience condition (Dutch: *M* = 3.19, *SD* = 1.04 vs. Moroccans: *M* = 4.13, *SD* = 0.92), *F*(1, 90) = 26.75, *p* < .001, η^2^ = .23. Furthermore, there was a near significant simple effect of mortality salience on the attitudes towards Dutch people: Participants were more positive in the mortality salience condition (*M* = 3.19, *SD* = 1.04) than in the control condition (*M* = 3.66, *SD* = 1.24), *F*(1, 90) = 3.77, *p* = .055, η^2^ = .04, but mortality salience had no effect on attitudes towards Moroccans (mortality salience condition: *M* = 4.13, *SD* = 0.92 vs. control condition: *M* = 4.13, *SD* = 1.06), *F* < 1, *ns*; η^2^ < .01. The effect of attitude target and the interaction between attitude target and mortality salience were not further qualified by an interaction with social category condition, both *F*s < 1, *ns*; η^2^ < .01.

#### Outgroup derogation

**Figure 2.**
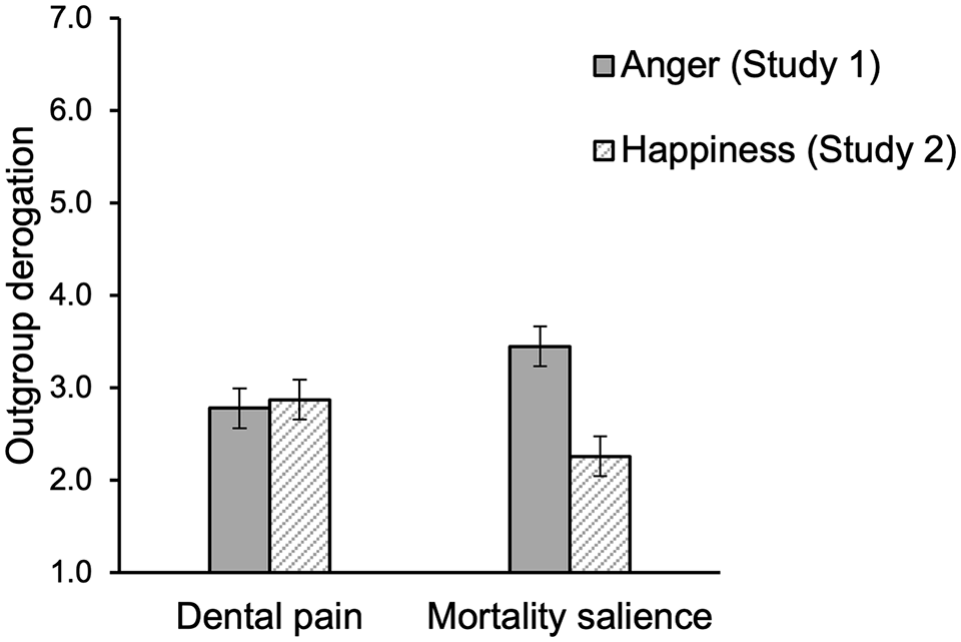
Outgroup derogation as a function of mortality salience in Study 1 (anger displays, dark grey bars) and Study 2 (happiness displays, light grey bars). *Note*. Error bars represent ±1 *SE*.

A 2 (mortality salience) x 2 (social category) ANOVA on the measure of outgroup derogation revealed a significant main effect of mortality salience, *F*(1, 90) = 4.28, *p* = .041, η^2^ = .05. When participants had viewed anger displays, they showed more outgroup derogation (i.e., agreed more with the statement that “the Koran is a violent and fascist book”) in the mortality salience condition (*M* = 3.45, *SD* = 1.54) than in the control condition (*M* = 2.77, *SD* = 1.56; see also Figure 2). The effect of social category condition and the two-way interaction between mortality salience and social category condition were both not significant, both *F*s < 1, *ns*, η^2^ < .01.

#### Facial behavior

We analyzed facial behavior with 2 (mortality salience) x 2 (social category) ANOVAs on AU4 (frowning), AU6 (smiling), and AU12 (smiling) activity separately. No effects were found.

## Study 2

Study 1 revealed the “classic” mortality salience effect that mortality salience increases worldview defense in such a way that attitudes towards the ingroup tended to become more positive and that outgroup derogation increased. These effects were observed in a context where participants viewed anger displays of either ingroup or outgroup models. The social category of the displayer did not further influence this effect, so there was no support for our prediction that the effect of mortality salience on worldview defense would be more pronounced when participants viewed outgroup anger displays. In Study 2, we investigated whether mortality salience would increase affiliation need in a context where participants viewed happiness displays.

### Method

Study 2 was identical to Study 1 in design, materials, and procedure, except for the emotion displays presented to participants. The study had a 2 (mortality salience: experimental vs. control) x 2 (social category: ingroup vs. outgroup) between-subjects design. We planned to collect data of 25 participants per group to make the study comparable to Study 1. In Study 2, there were 100 participants (55 female; *M*_age_ = 21.97, *SD*_age_ = 4.74), with 94 participants in the final sample after exclusions.^3^ A sensitivity analysis revealed that with a significance level of α = .05 and a power of (1 − β) = .80, this was sufficient for an effect size of *f* = .29.

### Results

#### Disclosure statement

Supplemental Material S2 presents the findings of a “thermometer” measure of group favorability, model ratings, and self-reported emotions (see also Endnote 2).

#### Manipulation checks

Paired *t* tests revealed that Northern European models were perceived as more native Dutch (*M* = 5.79, *SD* = 0.89) than the Mediterranean models (*M* = 2.91, *SD* = 0.82), *t*(93) = 19.68, *p* < .001, η^2^ = .81, and that Dutch participants perceived more overlap with Dutch people (*M* = 5.15, *SD* = 1.30) than with Moroccans (*M* = 2.63, *SD* = 1.24), *t*(93) = 15.05, *p* < .001, η^2^ = .71, showing that the manipulation of social category was successful. Furthermore, a paired *t* test revealed that participants perceived more happiness (*M* = 5.62, *SD* = 0.87) than anger (*M* = 1.89, *SD* = 0.82) in the emotion displays, *t*(93) = −26.02, *p* < .001, η^2^ = .88, demonstrating that the emotion displays were identified correctly.

#### Attitudes

A 2 (mortality salience, between-subjects) x 2 (social category, between-subjects) x 2 (attitude target: Dutch vs. Moroccan, within-subjects) mixed ANOVA on the measure of positive and negative thoughts revealed a significant main effect of attitude target, *F*(1, 90) = 11.95, *p* < .001, η^2^ = .12. Overall, Dutch participants thought more positively about Dutch people (*M* = 3.61, *SD* = 1.24) than about Moroccans (*M* = 4.10, *SD* = 1.18).

The two-way interaction between mortality salience and attitude target that was observed in Study 1, when participants observed anger displays, was not significant in Study 2 when participants viewed happiness displays, *F*(1, 90) = 1.53, *p* = .22, η^2^ = .02. However, simple effects analyses revealed that whereas attitudes towards Dutch people were more positive (*M* = 3.68, *SD* = 1.35) than attitudes towards Moroccans (*M* = 4.34, *SD* = 1.32) in the control condition, *F*(1, 90) = 11.02, *p* = .001, η^2^ = .11, this effect of attitude target was not significant in the mortality salience condition (Dutch: *M* = 3.53, *SD* = 1.12 vs. Moroccans: *M* = 3.85, *SD* = 0.98), *F*(1, 90) = 2.46, *p* = .12, η^2^ = .03. Furthermore, mortality salience had a significant effect on attitudes towards Moroccan people: Participants were more positive in the mortality salience condition (*M* = 3.85, *SD* = 0.98) than in the control condition (*M* = 4.34, *SD* = 1.32), *F*(1, 90) = 4.20, *p* = .043, η^2^ = .05. Mortality salience had no significant effect on attitudes towards Dutch people (mortality salience condition: *M* = 3.53, *SD* = 1.12 vs. control condition: *M* = 3.68, *SD* = 1.35), *F* < 1, *ns*; η^2^ < .01 (see Figure 1, bottom panel).

The two-way interaction between attitude target and social category, *F*(1, 90) = 1.53, *p* = .22, η^2^ = .02, and the three-way interaction between attitude target, social category, and mortality salience were also not significant, *F*(1, 90) = 1.66, *p* = .20, η^2^ = .02.

#### Outgroup derogation

A 2 (mortality salience) x 2 (social category) ANOVA on the measure of outgroup derogation revealed a significant main effect of mortality salience, *F*(1, 90) = 4.69, *p* = .033, η^2^ = .05, but in the opposite direction as the effect observed in Study 1: In Study 2, when participants viewed happiness displays, there was less outgroup derogation (i.e., agreed less with the statement that “the Koran is a violent and fascist book”) in the mortality salience condition (*M* = 2.26, *SD* = 1.24) than in the control condition (*M* = 2.87, *SD* = 1.47; see also Figure 2). The effect of social category condition and the two-way interaction between mortality salience and social category condition were both not significant, *F*s < 1, *ns*; η^2^ < .01.

#### Facial behavior

We analyzed facial behavior with 2 (mortality salience) x 2 (social category) ANOVAs on AU4 (frowning), AU12 (smiling), and AU6 (smiling) activity separately. No effects were found for AU4 and AU12. There was a marginal significant interaction between mortality salience and social category condition for AU6, *F*(1, 85) = 3.15, *p* = .080, η^2^ = .04. Follow-up simple effects analyses revealed that there was some indication that participants in the mortality salience condition smiled more in response to outgroup happiness displays (*M* = 2.05, *SD* = 3.46) than to ingroup happiness displays (*M* = 0.65, *SD* = 1.30), *F*(1, 85) = 3.59, *p* = .061, η^2^ = .04.

### Discussion

The findings of Studies 1 and 2 provide some initial support for the hypothesis that emotional signals regulate whether participants respond with worldview defense or a general affiliation tendency after reminders of death. In both Studies 1 and Study 2, Dutch participants had a more positive attitude towards Dutch than towards Moroccan people in the control condition. However, whereas the difference in attitudes towards Dutch and Moroccans was more pronounced in the mortality salience condition when participants had viewed anger displays (Study 1), the difference in attitudes was not observed in the mortality salience condition when participants had been exposed to happiness displays (Study 2). Furthermore, outgroup derogation was enhanced by mortality salience when participants had been exposed to anger displays (Study 1), but outgroup derogation was reduced when participants had been exposed to happiness displays (Study 2). These findings are in line with the proposition that mortality salience elicits worldview defense in response to emotions that signal distancing (i.e., anger), whereas it elicits affiliation need in response to emotions that signal affiliation (i.e., happiness).

It is interesting to note that the effects of mortality salience on ingroup favoritism and outgroup derogation were not moderated by whether the displays came from ingroup or outgroup expressers. This suggests that the emotion context alone (anger vs. happiness) is sufficient to determine effects of mortality salience in interpersonal encounters, independent of whether the source of the emotion is an ingroup or outgroup member.

## Study 3

The aim of Study 3 was to replicate and extend the findings of Study 1 and Study 2, and to make some methodological refinements. In Study 1 and Study 2, we filmed participants’ facial responses to the emotional display videos and coded their facial behavior, but none of the findings reached conventional levels of significance. One possibility is that FACS coding may not have been sensitive enough to pick up on subtle differences in facial activity between conditions. In Study 3, we therefore measured facial responses to the emotion displays with facial electromyography (EMG). Facial EMG provides a more precise measure of facial responses and is capable of picking up even subtle facial behavior (e.g., Dimberg, 1990). We assessed activity of the zygomaticus major and the orbicularis oculi to measure positive facial reactions, and of the corrugator supercilii to measure negative facial reactions (Dimberg, 1990; Hess & Fischer, 2013).

Furthermore, rather than investigating the effects of different emotion displays in separate studies, in Study 3, the type of emotion displays was part of the experimental design as a between-subject factor. Also, in addition to anger and happiness displays, we added sadness as a third emotion in the design. Like anger, sadness is an emotion with negative valence, but unlike anger, it lacks a threatening component (Aronoff et al., 1988, 1992). Like happiness, sadness is an emotion that signals need for affiliation (Fischer & Manstead, 2008), but unlike happiness, responsiveness to sadness has potential social costs as it can signal that one empathizes with the other’s sorrow and wants to ease it by providing succor (Bourgeois & Hess, 2008). Inclusion of sadness therefore enabled us to decompose responses to negative displays that are threatening from negative displays that are nonthreatening, and signals of affiliation without social costs from signals of affiliation with potential social costs.

Furthermore, in Study 3, social category of the expresser was introduced as a within-participant factor so we could compare both self-report and facial reactions to ingroup and outgroup displays directly. In addition, because ingroup and outgroup attitudes and outgroup derogation were measured with self-created, single-item response scales in Studies 1 and 2, we aimed to improve the measurement in Study 3 by using more reliable scales. We included a measure of perceived overlap with the model (the Inclusion of Other in the Self Scale [IOS]; Aron et al., 1992) as a self-report measure of affiliation, which was assessed before and after the presentation of stimuli. This enabled us to control for baseline ingroup biases in affiliation that may exist before any mortality salience and emotion manipulation, and to investigate the change in affiliation with ingroup and outgroup members.

Our hypotheses and predictions for anger and happiness displays were the same as in Study 1 and Study 2: We hypothesized that mortality salience would elicit worldview defense when individuals are exposed to anger displays, and that this would be more pronounced when exposed to outgroup displays; we further hypothesized that mortality salience would elicit affiliation need when individuals are exposed to happiness displays, independent of social categorization of the expresser. For sadness displays, we hypothesized that mortality salience would enhance affiliation need when exposed to ingroup displays, but not when exposed to outgroup displays.

For the facial behavior measure, we predicted that under mortality salience, participants would show more negative facial reactions in response to outgroup anger displays than to ingroup anger displays; more positive facial reactions to both ingroup and outgroup happiness displays compared to control; and more positive facial reactions to ingroup sadness displays compared to outgroup sadness displays. For the self-report measure of affiliation, we predicted that under mortality salience, perceived overlap with the ingroup would increase and perceived overlap with the outgroup would decrease after anger displays; that perceived overlap with both ingroup and outgroup would increase after happiness displays, when compared to control; and that perceived overlap with the ingroup would increase after sadness displays, but not with the outgroup.

### Method

#### Disclosure statement

The following sections provide a brief overview of the materials and procedure. For a more comprehensive overview of the methodology, see Supplemental Material S3.

#### Participants and design

Due to the resource intensive character of the EMG study, we planned to collect data of a minimum of 25 participants per group (150 overall). We ended up recruiting 157 participants in total (121 female, 36 male; *M*_age_ = 24.41, *SD*_age_ = 8.05, age range: 17–61 years), who participated in the study for a monetary reward (€7.00, about US$7.60 at the time). Participants were randomly assigned to condition in a 3 (emotion: anger vs. happiness vs. sadness, between-subjects) x 2 (mortality salience: control vs. experimental, between-subjects) x 2 (social category: ingroup vs. outgroup, within-subjects) mixed design. A sensitivity analysis revealed that with a significance level of α = .05, a power of (1 − β) = .80, and a correlation of within factors of *r* = .53 (correlation between ingroup and outgroup IOS change scores), the sample was sufficient to find a minimum effect size of *f* = .14. The study was approved by the Ethics Committee of the University of Salzburg.^4^

#### Materials and procedure

After giving informed consent, the experimenter cleaned participants’ skin and placed the EMG electrodes on the left side of the face on the zygomaticus major (associated with smiling), the orbicularis oculi (associated with smiling), and the corrugator supercilii (associated with frowning) muscle sites, following the guidelines of Fridlund and Cacioppo (1986). Participants then began the computerized task run by Inquisit 4.0 (Millisecond), starting with a premeasure of the IOS scale (Aron et al., 1992). Here, participants indicated how much overlap they felt between themselves and eight randomized neutral ADFES models (four Northern European and four Mediterranean models; two males and two females from each ethnic group). Because our participants were mainly of Central European nationalities (Austrian or German), the Northern European models served as ingroup members, whereas the Mediterranean models served as outgroup members. Next, participants engaged in the same mortality salience manipulation procedure as in Study 1 and Study 2 (Greenberg et al., 1990), and reported their current affective state using a German version of the Positive and Negative Affect Schedule (PANAS; Krohne et al., 1996; Watson et al., 1988) as a delay task (Burke et al., 2010). Participants were then exposed to anger, happiness, or sadness displays of the same eight ADFES models. The video clips were presented in two separate blocks (counterbalanced across participants), one with the ingroup and the other with the outgroup models. Participants reported their current affective state after each block. After both blocks, the IOS postmeasure of perceived overlap with the eight models was administered. Electrodes were removed and participants completed a questionnaire containing exploratory measures and demographic variables. Participants were then debriefed and received course credit or payment.

### Results

#### Disclosure statement

Detailed procedures of the EMG data preparation are presented in Supplemental Material S4. Results of measures concerning liking of the models; self-reported affect; Tajfel matrices; and perceived overlap of self, ingroup, and outgroup are reported in Supplemental Material S5 (see also Endnote 2).

#### EMG data preparation

EMG data were prepared according to standard procedures in EMG research (e.g., Fridlund & Cacioppo, 1986). Movement artefacts (e.g., blinks, swallowing) were removed from the signal; it was band-pass filtered between 30 and 300 Hz and with a 50 Hz notch filter, rectified, segmented, averaged into baseline (−2s−0s before each video onset), and averaged into five trial segments for each second of the video presentation (0s−1s, 1s−2s, 2s−3s, 3s−4s, and 4s−5s) with BrainVision Analyzer Version 2.1 (Brain Products, Gilching, Germany). We then averaged the eight baseline scores for the zygomaticus (α = .87), orbicularis (α = .93), and corrugator (α = .97) muscles (Russell, 1990).^5^

**Figure 3.**
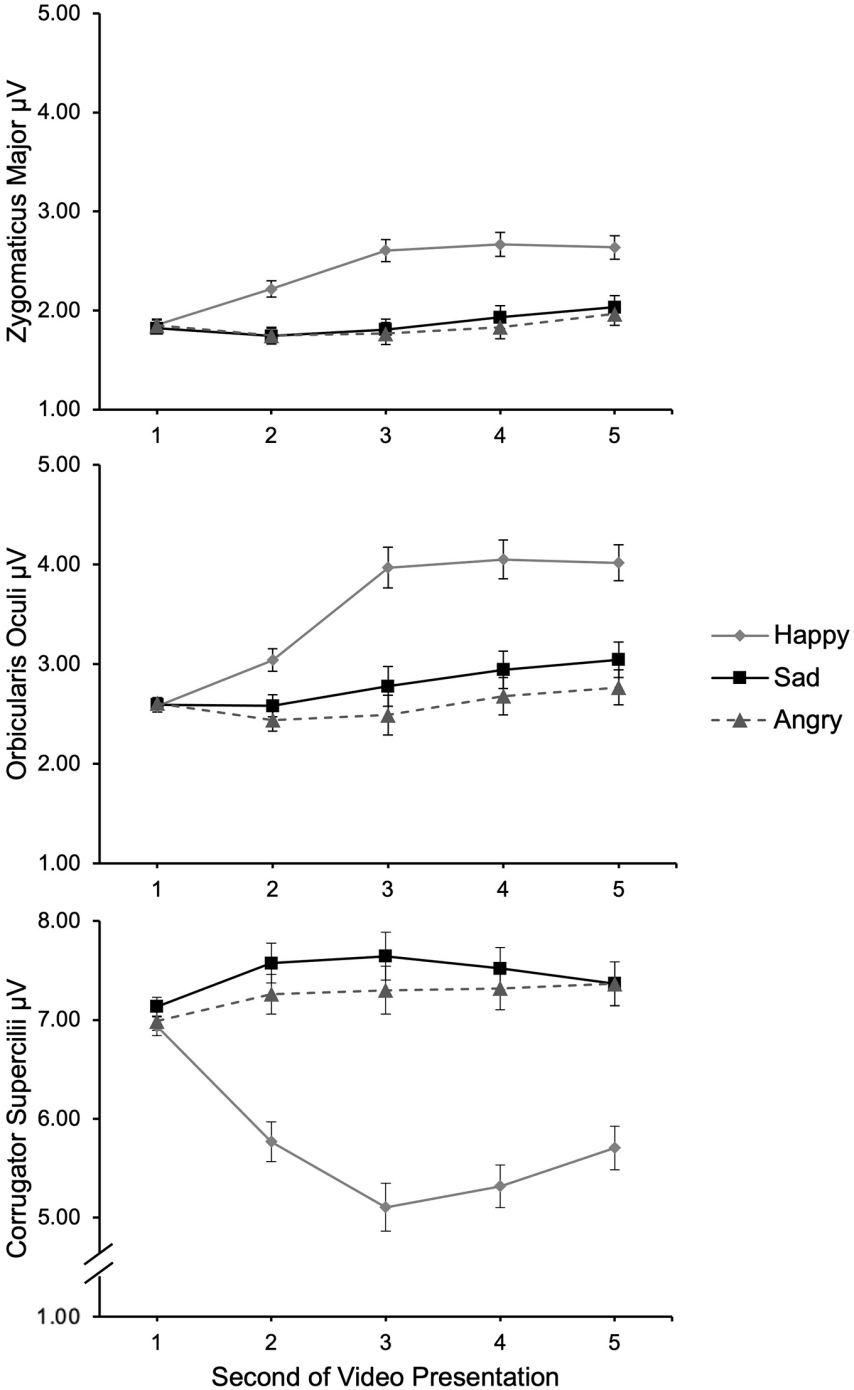
Muscle activity (zygomaticus major, A; orbicularis oculi, B; corrugator supercilii, C) as a function of emotion condition for each second of stimulus presentation. *Note*. Error bars represent ±1 *SE*.

Figure 3 presents muscle activity averaged across all models for each second of video presentation. As the figure shows, participants’ facial reactions were affected by emotion display after 2s of video presentation: There was more zygomaticus and orbicularis activity in response to happy facial expressions than to anger and sadness expressions, and (conversely) more corrugator activity in response to anger and sadness expressions than to happiness expressions. To test our hypotheses, we averaged muscle activity values between 2s and 5s of stimulus presentation.

#### Facial behavior

To test our hypotheses, we conducted 2 (emotion) x 2 (mortality salience) x 2 (social category) ANCOVAs on each muscle with the respective baseline value of each muscle as the covariate. Analysis of the corrugator, *F*(2, 145) = 1.94, *p* = .15, η_p_^2^ = .03, and orbicularis, *F* < 1, *ns*, did not reveal the predicted three-way interaction, but analysis of the zygomaticus revealed a significant three-way interaction between emotion, mortality salience, and social category, *F*(2, 143) = 5.93, *p* = .003, η_p_^2^ = .08. To decompose this interaction, we conducted follow-up 2 (mortality salience) x 2 (social category) ANCOVAs on the zygomaticus muscle (smiling) for each emotion condition separately. Descriptive values for the zygomaticus are depicted in Table 1.

**Table 1.** Means and standard deviations of zygomaticus muscle activity (not baseline corrected) and IOS change score as a function of mortality salience and emotion conditions.

	Control		Mortality salience	
	*M*	*SD*	*M*	*SD*
Anger displays				
Zygomaticus ingroup	1.50	0.79	1.83	1.01
Zygomaticus outgroup	1.53	0.94	1.42	0.46
IOS ingroup change	−0.09	0.63	0.29	0.50
IOS outgroup change	0.04	0.42	0.07	0.53
Happiness displays				
Zygomaticus ingroup	3.03	1.97	2.35	1.32
Zygomaticus outgroup	2.68	1.47	3.11	2.54
IOS ingroup change	0.61	0.73	0.32	0.52
IOS outgroup change	0.29	0.53	0.36	0.64
Sadness displays				
Zygomaticus ingroup	2.01	0.92	1.79	1.05
Zygomaticus outgroup	2.01	1.14	1.79	1.05
IOS ingroup change	−0.04	0.97	0.26	0.42
IOS outgroup change	0.01	0.72	0.17	0.45

*Note*. IOS = Inclusion of Other in the Self Scale.

**Figure 4.**
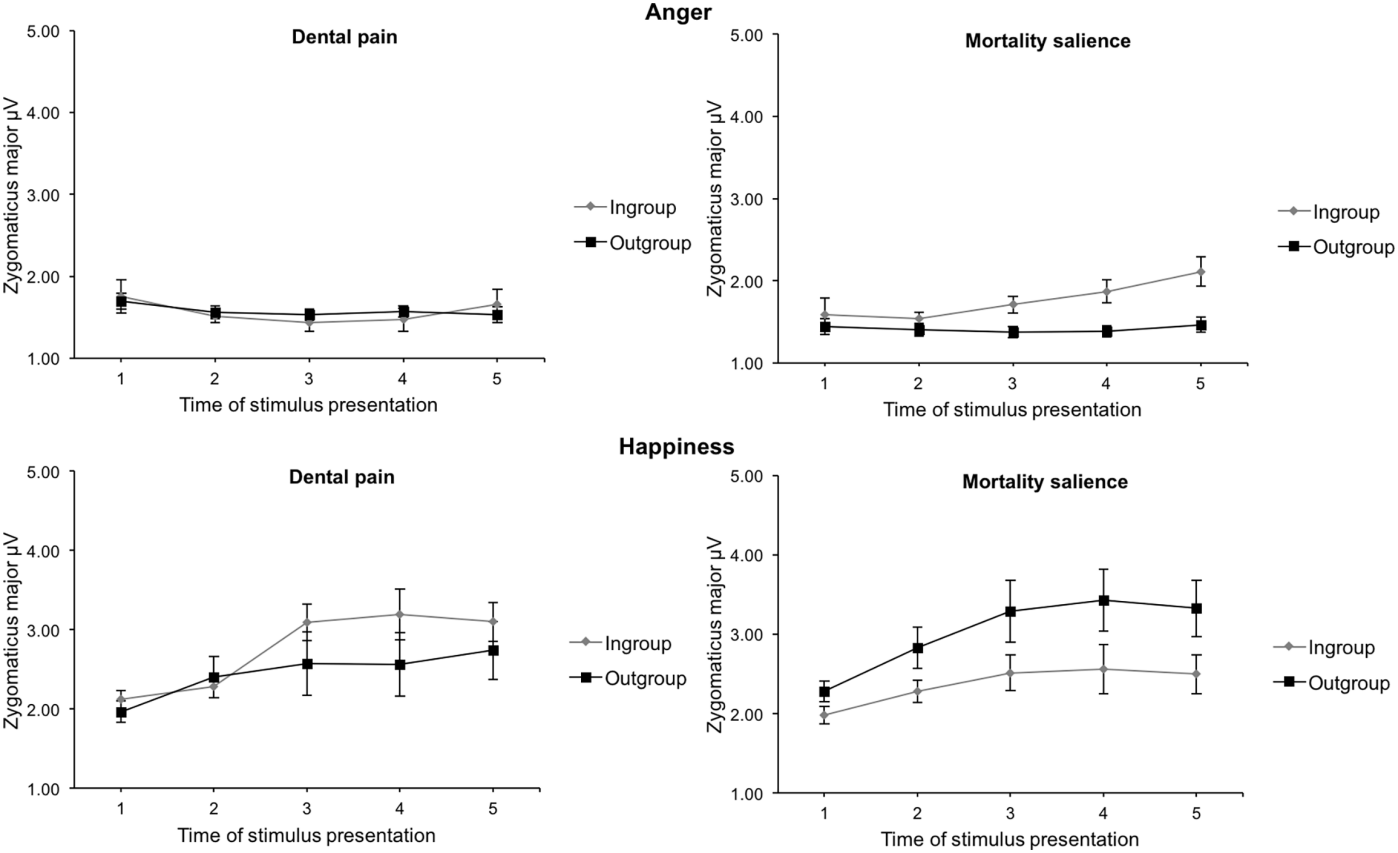
Zygomaticus major muscle activity in response to ingroup and outgroup anger (top panel, A) and happiness (bottom panel, B) displays as a function of time (stimulus presentation, in seconds) and mortality salience condition in Study 2. *Note*. Error bars represent ±1 *SE*.

There was a significant interaction between mortality salience and social category in the anger condition for zygomaticus activity, *F*(1, 47) = 4.81, *p* = .03, η_p_^2^ = .09. Participants did not differ on zygomaticus activity in response to ingroup and outgroup anger displays in the control condition, *F* < 1, *ns*, but responded with higher zygomaticus activity in response to ingroup anger displays than in response to outgroup anger displays in the mortality salience condition, *F*(1, 47) = 8.36, *p* = .006, η_p_^2^ = .15. In the happiness condition too, a significant two-way interaction between mortality salience and social category emerged, *F*(1, 46) = 5.32, *p* = .03, η_p_^2^ = .10. Muscle activity towards ingroup and outgroup happiness displays did not differ in the control condition, *F*(1, 46) = 1.04, *p* = .31, but participants reacted with more zygomaticus activity in response to outgroup happiness displays than in response to ingroup happiness displays in the mortality salience condition, *F*(1, 46) = 5.11, *p* = .03, η_p_^2^ = .10. In the sadness condition, the interaction between mortality salience and social category condition was not significant, *F* < 1, *ns*. Figure 4 depicts zygomaticus activity in response to ingroup and outgroup anger and happiness displays as a function of time and mortality salience condition.

In sum, participants who had been reminded of their mortality smiled more in response to ingroup anger than to outgroup anger, and more in response to outgroup happiness than to ingroup happiness.

#### Perceived overlap

For the IOS scale, a 2 (time: premeasurement vs. postmeasurement, within-subjects) x 3 (emotion, between-subjects) x 2 (mortality salience, between-subjects) x 2 (social category, within-subjects) mixed ANOVA revealed a significant four-way interaction, *F*(2, 151) = 4.23, *p* = .016, η_p_^2^ = .05.^6^ To decompose the interaction, we conducted three separate 2 (time: premeasurement vs. postmeasurement, within-subjects) x 2 (mortality salience, between-subjects) x 2 (social category, within-subjects) ANOVAs, one for each emotion condition.

**Figure 5.**
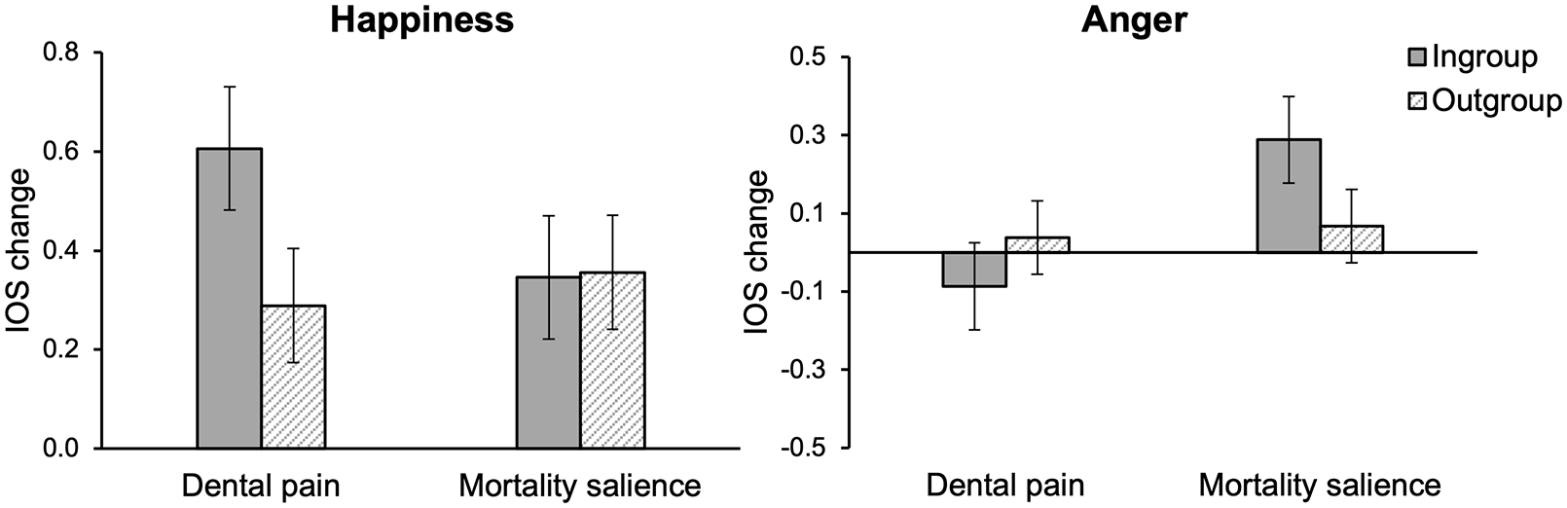
Change in perceived overlap with ingroup and outgroup targets (the difference between pre- and postmeasurement, within-subjects) as a function of mortality salience (between-subjects) and social category condition (within-subjects) for happiness (left) and anger (right): Study 3. *Note*. Error bars represent ±1 *SE*.

In the anger condition, there was a significant three-way interaction between time, mortality salience, and social category, *F*(1, 50) = 5.53, *p* = .02, η_p_^2^ = .10. In the happiness condition, the same three-way interaction between time, mortality salience, and social category did not reach conventional levels of significance, but was trending towards significance, *F*(1, 50) = 3.05, *p* = .087, η_p_^2^ = .06. In the sadness condition, the three-way interaction between time, mortality salience, and social category was not significant, *F* < 1, *ns*. To further decompose the three-way interactions in the anger and happiness conditions, we focused on the difference between the pre- and postmeasurements (change in perceived overlap, within-subjects), and investigated the effect of mortality salience and social category conditions. Results are displayed in Figure 5, and descriptive values for IOS change scores are depicted in Table 1.

When viewing anger displays, participants’ perceived overlap with both ingroup and outgroup members changed to the same extent in the control condition, *F*(1, 50) = 1.44, *p* = .24, but in the mortality salience condition, participants’ perceived overlap with ingroup targets increased more than with outgroup targets, *F*(1, 50) = 4.52, *p* = .04, η_p_^2^ = .08. When viewing happiness displays, participants’ perceived overlap with ingroup targets increased significantly more than their perceived overlap with outgroup targets in the control condition, *F*(1, 50) = 5.75, *p* = .02, η_p_^2^ = .10, but change in perceived overlap with ingroup and outgroup targets did not differ in the mortality salience condition, *F* < 1, *ns*.

It should be noted that, although Figure 5 may seem to suggest that — compared to the control condition — mortality salience decreased perceived overlap with the ingroup in the happiness condition (left panel of Fig. 5), this is due to the fact that the change score represents a relative increase in perceived overlap from pre- to postmeasurement. In fact, on the postmeasurement of perceived overlap, a main effect of social category indicated that the overlap with ingroup targets was higher than with outgroup targets.

To summarize, participants who had been reminded of their mortality and had been exposed to anger displays showed a greater increase in perceived overlap with ingroup than with outgroup targets. For participants who had been exposed to happiness displays, there was a greater increase in felt overlap with the ingroup than with the outgroup in the control condition, but in the mortality salience condition, the change in perceived overlap with ingroup and outgroup was similar.

### Discussion

In Study 3, we improved the measurement of facial reactions by using electromyography. We also included a validated scale of perceived overlap between self and other (IOS) as a measure of affiliation with ingroup and outgroup targets. In line with predictions, participants smiled more in response to anger displays of ingroup members than of outgroup members under mortality salience (as indicated by zygomaticus activity). In contrast, participants in the mortality salience condition smiled more in response to outgroup happiness displays than to ingroup happiness displays (we will turn to this pattern of findings in the General Discussion section).

Furthermore, participants who were reminded of their mortality and saw anger displays showed an increase in perceived overlap with the ingroup. This supports our hypothesis that mortality salience increases worldview defense after anger displays. In addition, whereas in the control condition perceived overlap with the ingroup increased more than perceived overlap with the outgroup after seeing happiness displays, participants who were reminded of their mortality showed similar increases in perceived overlap with ingroup and outgroup targets after seeing happiness displays. This suggests that, under neutral circumstances, happiness displays have a more positive impact on the evaluation of the ingroup compared to the outgroup, but this bias favoring the ingroup is absent under mortality salience.

For sadness displays, we hypothesized that mortality salience would enhance affiliation with one’s own social group, but not with the outgroup. However, perceived overlap with the ingroup did not increase more than perceived overlap with the outgroup in either the control or the mortality salience condition, and there were no effects of mortality salience and social category conditions on measures of facial behavior. It is possible that corrugator, zygomaticus, and orbicularis may not have been the appropriate muscles to measure specific facial responses to sadness. Perhaps measurement of activity in other muscles that are more uniquely associated with sadness (e.g., frontalis) would have provided a better assessment of facial reactions to sadness displays.

## General Discussion

We conducted three studies to test the idea that emotional displays influence whether individuals respond to mortality salience with worldview defense or a general tendency to affiliate with others. Across studies, we found that, when mortality was salient, participants who viewed anger displays showed more outgroup derogation and a tendency for more ingroup favoritism (Study 1), showed increased perceived overlap with the ingroup (Study 3), and displayed more positive facial reactions towards the ingroup versus the outgroup (Study 3). Taken together, these findings suggest that, under mortality salience, people feel the need to strengthen their ingroup ties and move away from outgroup members when confronted with displays that signal distancing—a replication of classical findings of worldview defense in studies of terror management theory (e.g., McGregor et al., 1998; Rosenblatt et al., 1989). In contrast, when mortality was salient and participants were exposed to happiness displays, ingroup favoritism and outgroup derogation decreased (Study 2), and participants showed more positive facial behavior to outgroup happiness (Study 3), demonstrating that mortality salience can bolster the need for affiliation with other groups when exposed to facial displays that signal an intention to foster social relations.

Previous research on terror management has also demonstrated that reminding people of their mortality not only enhances worldview defense but also the need to affiliate (e.g., Mikulincer et al., 2003; Taubman Ben-Ari et al., 2002; Wisman & Koole, 2003). Studies have identified cultural worldviews that promote tolerance between groups, prosocial norms, and individual differences in empathy as potential situational moderators that facilitate affiliation need (Greenberg et al., 1992; Jonas et al., 2008; Schimel et al., 2006). To our knowledge, the present research is the first to show that the effects of mortality salience can be moderated by lower level cognitive processes—that is, automatic responses to emotions. In addition, the finding that displays of happiness can improve outgroup attitudes under mortality salience adds to research showing increased sensitivity to emotionally evocative stimuli after mortality salience (Holbrook et al., 2011) and increased attention towards positive affective information under mortality salience—presumably as a coping mechanism to deal with the overwhelming terror associated with one’s prospective demise (De Wall & Baumeister, 2007). The current research suggests that in a social context, this increased attention to positive signals may have the added benefit of strengthening the bond that someone feels with others outside one’s immediate social group.

The findings also extend knowledge on the social function of emotions (Fischer & Manstead, 2008). Happiness displays can signal affiliative intentions (Hess et al., 2000; Knutson, 1996; Martin et al., 2017), and past research shows that people mimic smiling outgroup members as much as they mimic smiling ingroup members (Bourgeois & Hess, 2008; van der Schalk, et al., 2011). In the current studies, participants who were reminded of their own mortality smiled more in response to outgroup than to ingroup happiness displays. This suggests that under mortality salience, participants are even more likely to return a signal of affiliation from someone outside their immediate social group than from someone from their ingroup. Previous research has demonstrated that mortality salience can bolster the process described in Allport’s (1954) contact hypothesis and Gaertner et al.’s (1993) common ingroup identity model: Establishing personal connections between individual group members can initiate a recategorization mechanism whereby individuals who could be seen as outgroup members are included in one’s social group (Giannakakis & Fritsche, 2011; Motyl et al., 2011). The current findings similarly suggest that positive emotional displays from outgroup members can fulfill the affiliation need that is triggered by mortality salience and provide an opportunity to enlarge one’s social ingroup. If outgroup happiness displays are seen as signals that outgroup members perceive the ingroup as part of an inclusive social group, then increased smiling in return to these displays is a way to affirm that one is ready include outgroup expressers into a bigger (superordinate) social group.

For anger, the pattern of results for facial behavior in Study 2 revealed that under mortality salience, participants showed more zygomaticus activity in response to ingroup anger displays. This suggests that, when reminded of death, smiling in response to others’ anger is a way to ameliorate others’ negative feelings, and that this is more pronounced in reaction to someone we feel close to. Previous research has shown that individuals smile in response to others’ anger displays in order to ameliorate the others’ negative feelings: Häfner and IJzerman (2011) found that participants spontaneously smiled in response to anger displays of their significant other (i.e., they “smiled away” the anger of the other). In the context of the current study, smiling in response to anger displays may have similarly been a communicative response that was aimed at appeasement of a close other.

Taken together, our findings for zygomaticus muscle activity suggest that participants smiled to communicate affiliation towards ingroup and outgroup members, in line with the proposed social function of smiles (Fischer & Manstead, 2008; Martin et al., 2017; Niedenthal et al., 2010). However, our findings allow no conclusions about whether the smiles were “genuine” as expressions of felt joy (Feldman Barrett et al., 2019). For the interpretation of the findings of the current studies, we do not think it is necessary to distinguish between “genuine” and “fake” smiles. Instead, we propose that the smiles served as signals of affiliation rather than reflections of joy or felt reward (Martin et al., 2017; Niedenthal et al., 2010; Rychlowska et al., 2021).

## Limitations and Future Research

The reported studies have a number of limitations and some of the findings need to be interpreted with some caution. Firstly, in Study 1 and Study 2, results for facial behavior were inconclusive. It is possible that FACS coding may not have been sensitive enough to detect slight differences in facial muscle activity between conditions. In Study 3, facial reactions were recorded with EMG, which is sensitive enough to pick up very small differences in muscle activation, in order to provide a more robust measure of facial behavior. Only zygomaticus activity supported our hypotheses in Study 3, whereas activity of both corrugator and orbicularis muscle sites was unaffected by mortality salience and social category conditions. Although EMG is a more sensitive and less subjective measure of facial activity, it can only measure activity of a limited number of muscle sites. As such, it does not capture the full plethora of facial behavior, and it is possible that certain other or idiosyncratic facial responses may have been missed.

Secondly, because Study 1 only presented anger displays and Study 2 only presented happiness displays, it was not possible to make a direct comparison between emotions across these studies. In addition, social category was a between-subjects factor in Study 1 and Study 2, which limited the power of these studies (Brysbaert, 2019), and (facial) responses to ingroup and outgroup displays could not be compared within participants. Study 3 manipulated emotion displays as a between-subjects factor and social category as a within-subjects factor, which revealed differential reactions to ingroup and outgroup anger and happiness displays that could be observed with more direct comparisons.

In the reported studies, there was also empirical evidence that did not provide support for the hypothesis that emotional displays moderate the effects of mortality salience. Other measures included in the current studies such as group favorability (Studies 1 and 2), liking of the models (Studies 1 and 2), self-reported affect (Studies 1 and 2), Tajfel matrices (Tajfel et al., 1971), and the OSIO scale (Study 3; Schubert & Otten, 2002) showed no significant effects of mortality salience (see supplemental material). This may be due to the fact that the reported effect sizes were small and the statistical power of the studies may not have been sufficient to detect possible effects on all dependent variables. As such, the current studies only provide suggestive evidence that emotions and terror management have a combined effect on worldview defense and affiliation need. It is important to note that FACS coding and EMG measurement are labor intensive, and that the sample sizes of the current studies were bigger than in comparable studies.

An important area for future research is to directly or conceptually replicate these findings, especially in light of current failure to replicate classical terror management findings (Klein et al., 2019; Rodríguez-Ferreiro et al., 2019). For this debate, the current findings are informative because they show that the emotional context in which mortality salience manipulations take place might be an important moderator of the effects of mortality salience, which may be relevant for the interpretation of different findings in the literature. Future research could also investigate reactions to other facial displays such as dominance, contempt, regret, and/or pride. Based on the findings reported here, it would be predicted that the specific social meaning conveyed by these displays would similarly moderate responses to reminders of mortality.

## Conclusion

In the current set of studies, we investigated whether the emotional context in which people are threatened by the thought of their own mortality determines worldview defense or a general affiliation tendency. Across studies, we found that, in the context of anger displays, ingroup attitudes became more positive and outgroup derogation increased, whereas in the context of happiness displays, outgroup derogation decreased and participants smiled more in response to outgroup happiness. Due to rather small effect sizes, we conclude that the current studies should be taken as preliminary evidence that displays of emotions influence whether relations between groups are likely to deteriorate or improve when mortality is salient. When facing existential threat, subtle emotional signals may be decisive for whether we embrace or exclude our fellow human beings.

## Supplemental Material

sj-docx-1-gpi-10.1177_13684302221128229 – Supplemental material for Existential threat and responses to emotional displays of ingroup and outgroup members Click here for additional data file. Supplemental material, sj-docx-1-gpi-10.1177_13684302221128229 for Existential threat and responses to emotional displays of ingroup and outgroup members by Janet Wessler, Job van der Schalk, Jochim Hansen, Johannes Klackl, Eva Jonas, Maurice Fons, Bertjan Doosje and Agneta Fischer in Group Processes & Intergroup Relations

## Notes

1. One participant was familiar with the theoretical background of the study and his/her answer to the mortality salience manipulation revealed that s/he may not have responded seriously; this person’s data were excluded. Five participants were excluded because of their ethnicity (Arabic/Middle Eastern). One participant was excluded because this person was limited in his/her ability to speak Dutch.

2. All three studies included several additional measures apart from those presented here. We present findings for measures that best operationalize our hypotheses and are most comparable between studies. Results for all other measures are presented in the supplemental material. These were either not related to the hypotheses, less comparable between studies, or had limited interpretability due to technical errors.

3. In Study 2, there was an error in the experimental program, which was corrected after the first two participants; data from these participants were subsequently removed from the analyses. Data from another participant were excluded because this person did not give a serious answer to the manipulation question. Data from three further participants were excluded because of their ethnicity (Arabic/Middle Eastern).

4. The majority of participants were German or Austrian citizens (*n* = 149), students (*n* = 125), and Christian (*n* = 117). There were seven participants in the sample who reported being of a nationality other than Austrian or German. Thirty-two participants reported having no religion, two participants reported being Jewish, one participant reported being Muslim, and four participants reported being of other religion. Data from all participants were retained.

5. Four participants were excluded from the EMG data analysis because of technical problems during data acquisition or bad data quality. In addition, we excluded baseline or trial values with muscle activity of z > 3.5. If a participant had three or more missing trial values in either the four ingroup or the four outgroup trials, we excluded this participant from EMG data analyses of that muscle (zygomaticus major: *n* = 3; orbicularis oculi: *n* = 1; corrugator supercilii: *n* = 1). For the remaining participants, the procedure resulted in exclusion of 1.25% of baseline and 0.92% of zygomaticus trials; 0.33% of baseline and 0.33% of orbicularis trials; and 0.16% of baseline and 0.08% of corrugator trials.

6. The ANOVA also revealed a significant main effect of social category, *F*(1, 151) = 28.83, *p* < .001, η_p_^2^ = .16; a significant main effect of time, *F*(1, 151) = 20.72, *p* < .001, η_p_^2^ = .12; a two-way interaction between social category and mortality salience, *F*(1, 151) = 4.08, *p* = .045, η_p_^2^ = .03; and a two-way interaction between time and emotion, *F*(1, 151) = 5.98, *p* = .003, η_p_^2^ = .07. These effects will not be further interpreted, as they are qualified by the significant four-way interaction.
