# Supplementary material for: Existential threat and responses to emotional displays of ingroup and outgroup members
Source: Group Process Intergroup Relat. 2022 Nov 5;26(8):1866–87. doi: 10.1177/13684302221128229 (PMC10665133; doi:10.1177/13684302221128229)
Supplement: sj-docx-1-gpi-10.1177_13684302221128229 – Supplemental material for Existential threat and responses to emotional displays of ingroup and outgroup members [file sj-docx-1-gpi-10.1177_13684302221128229.docx]

**Supplemental material**

**S1. Study 1 and Study 2– Complete methodology and procedure**

At the start of the study participants provided consent and were presented with the Emotional Contagion Scale (Doherty, 1997). Participants then received the mortality salience manipulation, followed by a delay task: the Interpersonal Reactivity Index (Davis, 1980). Following this, the main task started.

Before presenting the emotional displays we measured self-reported anger and happiness with four items that were embedded in a 17-item questionnaire that ostensibly measured ‘experimental stress.’ Participants rated their anger (“I feel angry”), irritation (“I feel irritated”), happiness (“I feel happy”) and cheerfulness (“I feel cheerful”) on a scale from 1 (*not at all*) to 7 (*completely*).

While viewing the emotional displays participants were unobtrusively filmed and facial behavior was FACS coded (Ekman & Friesen, 1978). Frequency and intensity of AU4 (brow lowerer, associated with frowning), AU6 (cheek raiser and eye lid compressor, associated with smiling), and AU12 (lip corner puller, associated with smiling) were scored and combined to create facial activity scores. When it was not possible to code facial behavior (e.g., when the face was not visible in the recordings), AU scores were entered as missing values. As a result, AU4 activity of six participants, AU6 activity of five participants, and AU12 activity of ten participants were not coded.

Following the presentation of the emotion displays the questionnaire measuring self-reported anger and happiness was administered for a second time. Next, participants rated the neutral expressions of both White and Mediterranean models on five personality measures (extraversion, friendliness, conscientiousness, emotional stability, and openness to experience). As a measure of liking participants rated the models on attractiveness (1 = *very unattractive*, 7 = *very attractive*), positivity-negativity (-3 = *negative*, 3 = *positive*), and likeability (1 = *very unlikeable*, 7 = *very likeable*).

As manipulation checks, participants indicated the extent to which the models seemed native Dutch or non-native Dutch; their perceived overlap with Dutch people and Moroccans, using the Overlap of Self Ingroup and Outgroup questionnaire (OSIO; Schubert & Otten, 2002); and their identification with native Dutch as a group (Leach et al., 2008).

Next, participants indicated the extent to which their impression about Dutch and Moroccan people was favorable on a ‘thermometer’ measure from 0° (*least favorable*) to 100° (*most favorable*) and the ingroup and outgroup attitude measures were administered. Following this, participants did a Single Category Implicit Association Test (Karpinski & Steinman, 2006) with Dutch and Arabic names as categories. Participants then indicated the extent to which they had perceived the models as groups or as individuals. As a manipulation check of emotion, participants indicated the perceived intensity of anger (Study 1) and joy (Study 2) in the stimuli they had been exposed to.

Finally, demographic information was collected. This included questions about participants’ political affiliations, the measure about the film on the Koran, and a measure on extent to which participants felt that the government should prohibit the broadcasting of the film. We also measured attitudes to Dutch military presence in Afghanistan. At the end of experiment participants were debriefed, and received course credit or were paid.

**S2. Study 1 – Additional results**

Here we report findings of manipulation checks, the thermometer measure, model ratings, and self-reported emotions.

***Thermometer measure***. A 2 (Attitude Target: Dutch vs. Moroccan, within-subjects) by 2 (Mortality Salience condition, between-subjects) by 2 (Social Category condition, between-subjects) mixed ANOVA on the thermometer measure revealed a significant effect of attitude target, *F*(1, 89) = 76.43, *p* < .001, η^2^ = .46. Dutch participants indicated more favorable impressions about Dutch (*M* = 69.47, *SD* = 10.93) than Moroccans (*M* = 55.10, *SD* = 16.37). No other effects were significant.

***Liking***. Due to a programming error, positivity and negativity ratings of the models for the first 31 participants was incorrectly labeled. For these participants answers on this item were treated as missing variables. Reliability analyses revealed that for both the ingroup and outgroup models Cronbach’s alpha was enhanced when the attractiveness items were excluded from the liking scale. We therefore averaged the positivity-negativity items and the likeability items to create a liking of ingroup models (*r* = .73) and a liking of outgroup models scale (*r* = .79).

A 2 (Model ethnicity, within-subjects) by 2 (Mortality Salience condition, between-subjects) by 2 (Social Category condition, between-subjects) mixed ANOVA on the liking measures only revealed a significant interaction between social category condition and model ethnicity, *F*(1, 62) = 5.57, *p* = .021, η^2^ = .08. Follow-up simple effects analyses revealed that liking of North-European models was not affected by whether people had been exposed to emotion displays of ingroup (*M* = 4.42, *SD* = 0.77) or outgroup (*M* = 4.41, *SD* = 0.59), *F* < 1, *ns*, η^2^ < .01, whereas liking of Mediterranean models was higher when people had been exposed to emotion displays of outgroup (*M* = 4.57, *SD* = 0.69) compared to when they had been exposed to emotion displays of the ingroup (*M* = 4.20, *SD* = 0.69), *F*(1, 62) = 4.81, *p* = .032, η^2^ = .07, suggesting that people are more positive about outgroup members when they have viewed their emotion displays. None of the effects involving the mortality salience manipulation were significant.

***Self-reported emotions.*** The anger and irritation items were combined to create an anger scale (*r_pre-measure_* = .59, *r_post-measure_* = .64). A 2 (Time: pre vs. post, within-subjects) by 2 (Mortality Salience) by 2 (Social Category) mixed ANOVA on the anger scale revealed a near significant main effect of time, *F*(1, 90) = 3.82, *p* = .054, η^2^ = .04, and a significant two-way interaction between time and social category condition, *F*(1, 90) = 5.86, *p* = .017, η^2^ = .06. Overall, self-reported anger tended to increase after viewing anger displays (*M_pre_* = 1.64, *SD_pre_* = 0.85; *M_post_* = 1.80, *SD_post_* = 1.07). However, the increase in self-reported anger was only present when participants had viewed ingroup displays (*M_pre_* = 1.66, *SD_pre_* = 0.85; *M_post_* = 1.99, *SD_post_* = 1.18; *F*(1, 90) = 9.79, *p* = .002, η^2^ = .10) and not when participants had viewed outgroup displays (*M_pre_* = 1.63, *SD_pre_* = 0.86; *M_post_* = 1.60, *SD_post_* = 0.92; *F* < 1, *ns*). This shows that emotional contagion was stronger in response to ingroup displays compared to outgroup displays (Van der Schalk et al., 2011). No main effect or interactions involving the mortality salience condition were found.

**S3. Study 2 – Additional results**

Here we report findings of manipulation checks, the thermometer measure, model ratings, and self-reported emotions.

***Thermometer measure***. A 2 (Attitude Target: Dutch vs. Moroccan, within-subjects) by 2 (Mortality Salience condition, between-subjects) by 2 (Social Category condition, between-subjects) mixed ANOVA on the thermometer measure revealed a significant effect of attitude target, *F*(1, 90) = 84.52, *p* < .001, η^2^ = .48. Dutch participants indicated more favorable impressions about Dutch (*M* = 69.11, *SD* = 12.60) than Moroccans (*M* = 55.67, *SD* = 18.07).

There also was a marginal significant two-way interaction between attitude target and mortality salience condition, *F*(1, 90) = 3.65, *p* = .059, η^2^ = .04. In the mortality salience condition Dutch participants indicated somewhat more favorable impressions about Moroccans (*M* = 58.06, *SD* = 17.54) than in the control condition (*M* = 53.28, *SD* = 18.46), but this was not significant, *F*(1, 90) = 1.59, *p* = .21, η^2^ = .02. No other effects were significant.

***Liking***. Reliability analyses revealed that for both the ingroup and outgroup models Cronbach’s alpha was enhanced when the attractiveness items were excluded from the liking scale. We therefore averaged the positivity-negativity items and the likeability items to create a liking of ingroup models (*r* = .72) and a liking of outgroup models scale (*r* = .79).

A 2 (Model ethnicity, within-subjects) by 2 (Mortality Salience condition, between-subjects) by 2 (Social Category condition, between-subjects) mixed ANOVA on the liking measures revealed a significant main effect of model ethnicity, *F*(1, 90) = 4.15, *p* = .045, η^2^ = .04, and a significant interaction between social category condition and model ethnicity, *F*(1, 90) = 9.91, *p* = .002, η^2^ = .10. Follow-up simple effects analyses revealed that liking of North-European models (*M* = 4.70, *SD* = 0.60) was higher than liking of Mediterranean models (*M* = 4.21, *SD* = 0.56) when participants had been exposed to emotion displays of ingroup, *F*(1, 90) = 13.45, *p* < .002, η^2^ = .13, whereas liking of North-European models (*M* = 4.49, *SD* = 0.61) and Mediterranean models (*M* = 4.47, *SD* = 0.91) did not differ significantly when participants had been exposed to emotion displays of outgroup, *F* < 1, *ns*. Viewed differently, liking of North-European models was somewhat higher when participants had been exposed to ingroup emotion displays (*M* = 4.70, *SD* = 0.60) compared to outgroup emotion displays (*M* = 4.49, *SD* = 0.61) , *F*(1, 90) = 2.86, *p* = .094, η^2^ = .03, whereas liking of Mediterranean models was somewhat higher when participants had been exposed to outgroup emotion displays (*M* = 4.47, *SD* = 0.91) compared to ingroup emotion displays (*M* = 4.21, *SD* = 0.56), *F*(1, 90) = 2.79, *p* = .098, η^2^ = .03, suggesting that viewing happiness displays of members of a particular group leads to more liking of members of that group. None of the effects involving the mortality salience manipulation were significant.

***Self-reported emotions.*** The happiness and cheerfulness items were combined to create a happiness scale (*r_pre-measure_* = .89, *r_post-measure_* = .95). A 2 (Time: pre vs. post, within-subjects) by 2 (Mortality Salience) by 2 (Social Category) mixed ANOVA on the happiness scale revealed a marginal significant two-way interaction between time and social category condition, *F*(1, 90) = 2.78, *p* = .099, η^2^ = .03: Self-reported happiness tended to increase after viewing outgroup happiness displays (*M_pre_* = 4.37, *SD_pre_* = 1.41; *M_post_* = 4.51, *SD_post_* = 1.51; *F*(1, 90) = 3.28, *p* = .074, η^2^ = .04), but not when participants had viewed ingroup happiness displays (*M_pre_* = 4.68, *SD_pre_* = 1.30; *M_post_* = 4.64, *SD_post_* = 1.37; *F* < 1, *ns*). This shows that emotional contagion was stronger in response to *outgroup* displays compared to ingroup displays. No effects involving the mortality salience condition were found.

**S4. Study 3 – Complete materials and procedure**

After giving informed consent (including agreement to the placement of facial sensors and the making of a video recording during the experiment), the experimenter cleaned participants’ skin and placed the facial electromyography (EMG) electrodes following the guidelines of Fridlund and Cacioppo (1986). Participants’ skin was cleansed with Nuprep (Weaver and Company) and 70% alcohol. The TMSi bipolar micro electrodes filled with Signa Gel (Parker Laboratories) were placed on the left side of the face on the Corrugator Supercilii (associated with frowning), the Orbicularis Oculi (associated with Duchenne smiling), and the Zygomaticus Major (associated with smiling) muscle sites. The raw EMG signal was sampled at 1024 Hz using a TMSi Refa 72 Stationary System (TMS international, Oldenzaal, The Netherlands) and was recorded with TMSi Polybench software.

Participants then began the computerized task (Inquisit software 4.0), starting with a pre-measurement of liking of and perceived overlap with eight ADFES models in random order. The stimuli depicted four North-European and four Mediterranean models (two male and two female for each ethnic group). For our sample, North-European models represented members of their own ethnic group (ingroup), whereas Mediterranean models represented members of a different ethnic group (outgroup). During this task, participants saw neutral videos of each model and, on the same page, indicated how much they liked the model on two items (“How much do you like this person?”, “How much would you like to meet this person?”) on a scale from 0 (not at all) to 6 (very much). As a measure of perceived similarity participants indicated how much overlap they felt between themselves and the model with the Inclusion of Other in the Self Scale (IOS; Aron, Aron, & Smollan, 1992).

Then, participants engaged in the same mortality salience priming procedure as in Study 1 and Study 2 (Greenberg et al., 1990). As a delay task, participants indicated their current affective state with a German version of the PANAS (Watson, Clark, & Tellegen, 1988; Krohne, Egloff, Kohlmann, & Tausch, 1996) and four additional exploratory items, that is: schadenfreude, compassion, surprise, and contempt.

In order to manipulate the emotional context, participants were exposed to anger, happiness, and sadness displays with face-forward video clips of the eight ADFES models. The video clips were presented in two separate blocks (counter-balanced across participants), one with the four ingroup models and one with the outgroup models. The inter-trial time between the videos was 10 seconds during which time a fixation cross appeared. After each block, participants rated how much they thought the models had expressed disgust, fear, joy, sadness, and anger on a scale from 0 (not at all) to 6 (very much) to check if participants correctly identified the respective emotion expressed (items measuring perceived disgust, surprise, and fear were included as control items). They also indicated their current affective state on the same PANAS items as in the delay task after each block. The items of the PANAS were averaged into a positive affect score (after priming, α = .80; after the ingroup stimuli, α = .86; and after the outgroup stimuli, α = .86), and a negative affect score (after priming, α= .85; after the ingroup stimuli, α = .91; and after the outgroup stimuli, α = .89).

After both blocks of emotion displays, the post measurement of liking and perceived overlap with the four ingroup and four outgroup ADFES models followed, using the same items as during the pre-measurement. We averaged the pre-measurement liking ratings across ingroup (α = .85) and outgroup (α = .90) models, and the post-measurement liking ratings across ingroup (α = .87) and outgroup (α = .89) models. For the IOS scale, we similarly averaged the pre-measurement ratings across ingroup (α = .80) and outgroup (α = .78) models, and the post-measurement ratings across ingroup (α = .82) and outgroup (α = .82) models.

Participants were then presented with six matrices as described by Tajfel, Billig, Bundy and Flament (1971). They were told that the task was to play a game in which they distribute monetary units between ingroup and outgroup members. Participants chose one out of 14 options in the matrix (14 being the choice of maximum reward possible for the ingroup member). Afterwards, we measured how participants perceived themselves relatively to the ingroup and outgroup with the OSIO scale (Schubert & Otten, 2002).

The electrodes were then removed and participants filled in a final questionnaire with exploratory measures as potential moderators. This included six items of the ethnocentrism-scale (Bizumic, Duckitt, Popadic, Dru, & Krauss, 2009), a ten-item German version of Rosenberg’s self-esteem scale (Rosenberg, 1965; Von Collani & Herzberg, 2003), and 12 items of the German version of the Interpersonal Reactivity Scale (empathetic concern, perspective taking, and fantasy subscale; Davis, 1980; Paulus, 2009).

Participants finally indicated their age, gender, profession or field of study, nationality, and religion. The experimenter orally asked about any suspicions participants had regarding the experimental procedure and the purpose of the study. No participant guessed the purpose correctly. At the end, participants were debriefed, received course credit or were paid, and dismissed.

**S5. Study 3 – Additional results**

In the following, we report results for measures of liking, self-reported affect, the Tajfel matrices, and the OSIO scale. When interpreting the results, please note that the Tajfel matrices and the OSIO scale were taken at the very end of the experiment, where we could no longer distinguish between the effects of ingroup and outgroup displays.

***Liking of the models.*** There was a significant four-way interaction between time (pre vs. post, within-subjects), emotion (between-subjects), social category (within-subjects), and mortality salience (between-subjects), *F*(2, 151) = 3.75, *p* = .026, η_p_^2^ = 0.05. To break down the interaction we investigated the effect of mortality salience and social category conditions by looking at the change in liking from pre- to post-measurement in each emotion condition.

When viewing anger displays, participants’ change in liking did not differ significantly as a function of social category in the control condition, *F*(1, 50) = 3.10, *p* = .08, η_p_^2^ = .06, but in the mortality salience condition participants’ change in liking of ingroup models increased significantly more than change in liking of outgroup models, *F*(1,50) = 4.63, *p* = .04, η_p_^2^ = .09. When viewing happiness displays, participants’ increase in liking of ingroup models was significantly greater than increase in liking of outgroup models in the control condition, *F*(1,50) = 6.62, *p* = .01, η_p_^2^ = .12, but change in liking of ingroup and outgroup models did not differ in the mortality salience condition, *F*(1,50) = 1.01, *ns*. In the sadness condition there were no significant effects, *F*s < 1. Results for anger and happiness conditions are displayed in Figure S5.


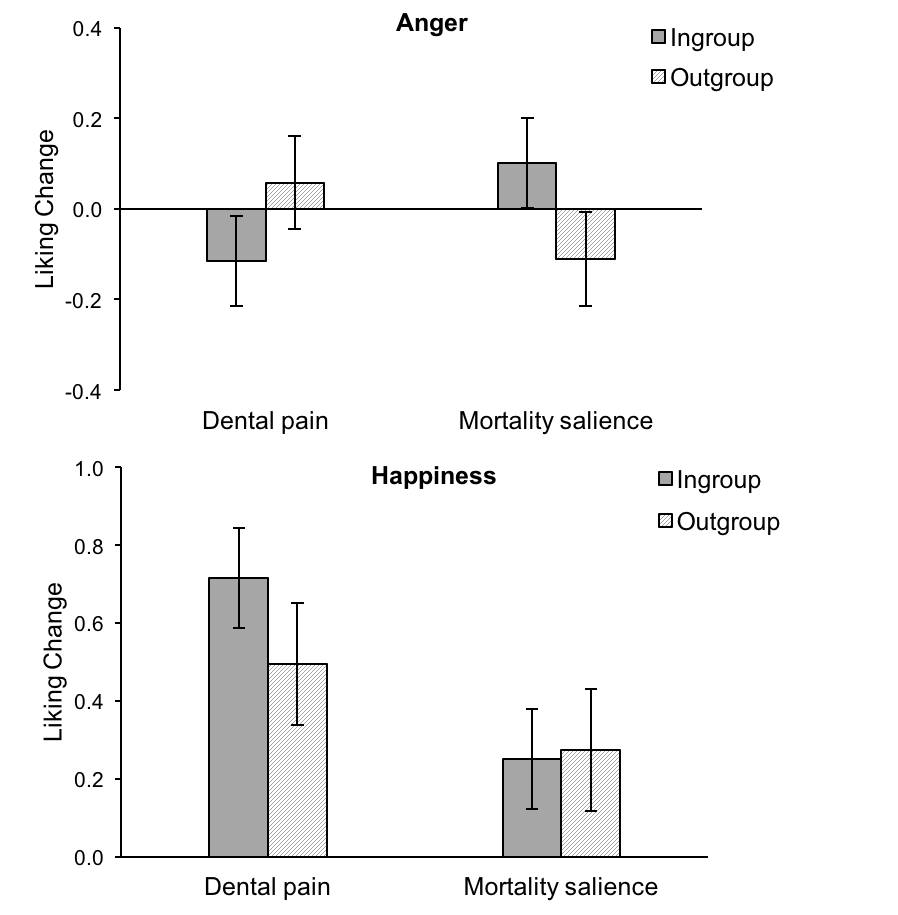


*Figure S5*. Change in liking of models (pre- vs. post-measurement) as a function of social category and mortality salience in the anger (top panel, A) and happiness (bottom panel, B) conditions in Study 2. Error bars represent ± 1 *SE*.

***Self-reported positive affect.*** For the positive affect scale a significant three-way interaction between emotion (between-subjects), mortality salience (between-subjects), and social category (within-subjects) conditions was found, *F*(2, 151) = 3.16, *p* = . 045, η_p_^2^ = 0.04. As follow-up we conducted three 2 (Mortality salience) x 2 (Social category) ANOVAs on positive affect, for each emotion condition separately.

In the happiness condition there was a significant interaction between mortality salience and social category , *F*(1, 50) = 10.36, *p* = .002, η_p_^2^ = .17 (see Figure S6): In the control condition, participants reported more positive affect after having seen ingroup displays, *F*(1, 50) = 9.65, *p* = .003, η_p_^2^ = .16. In the mortality salience condition, by contrast, there was no difference in positive affect between ingroup and outgroup conditions, *F*(1, 50) = 2.09, *p* = .16. In the anger, *F* < 1.10, *ns*, and sadness, *F* < 1.58, *ns*, conditions the interaction between mortality salience and social category were not significant. Results for the happiness condition are displayed in Figure S6.

*
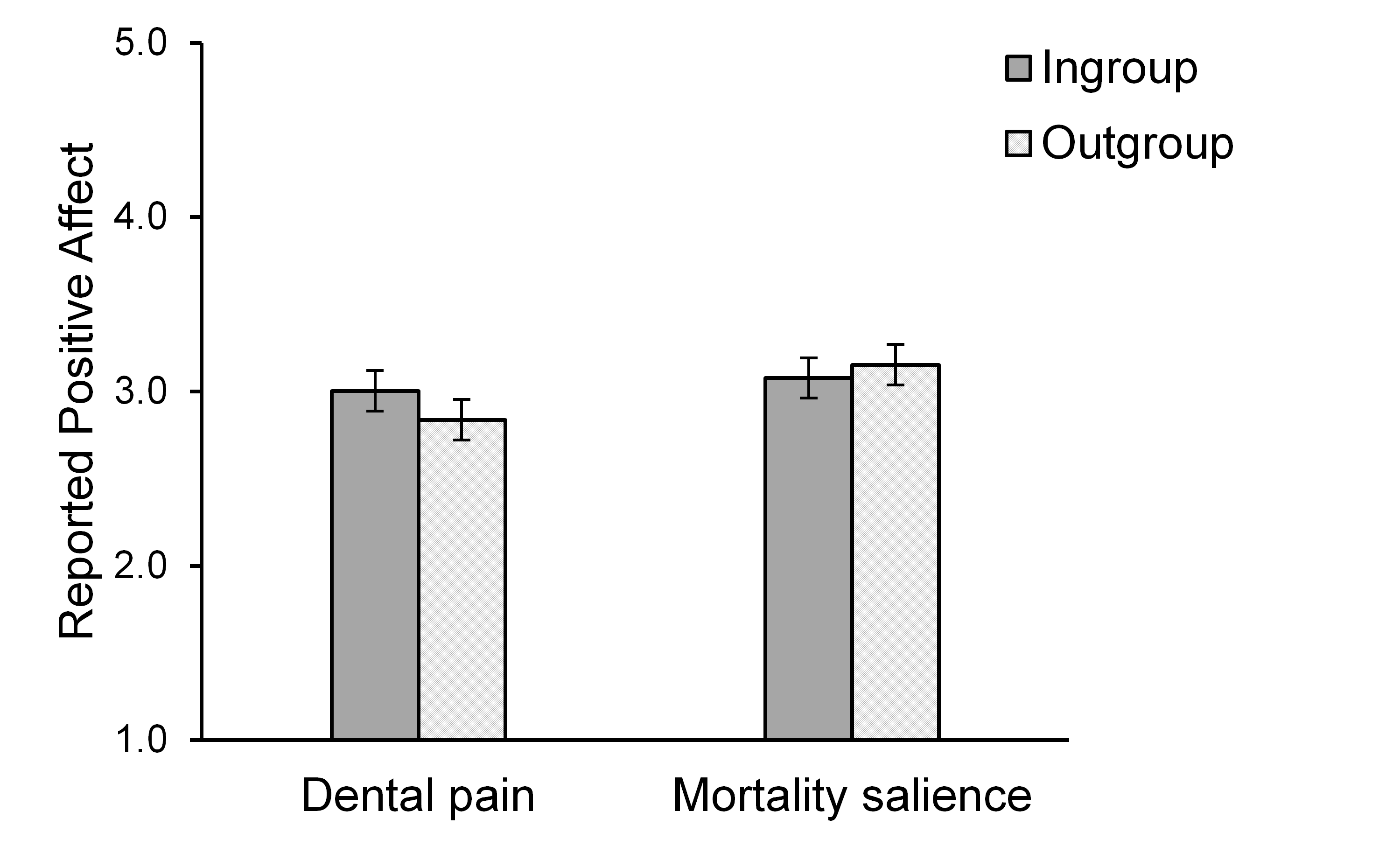
*

*Figure S6*. Self-reported positive affect as a function of social category and mortality salience in the happiness condition (Study 2). Error bars represent ± 1 *SE*.

***Self-reported negative affect.*** For the negative affect scale the three-way interaction between emotion, mortality salience and social category was not significant, *F*(2, 151) = 2.31, *p* = .103, η_p_^2^ = 0.03.

***Tajfel matrices.*** Participants scores on the Tajfel matrices were averaged (α = .78). A score > 7.5 indicates ingroup favoritism. We conducted a 3 (Emotion) by 2 (Mortality salience) between-participants ANOVA on the ingroup favoritism scores. There were no main effects, *F*s < 1, and the interaction was also not significant, *F*(2, 151) = 1.14, *p* = .32, η_p_^2^ = .02. We also performed a one-sample *t*-test in order to check if the choices on the matrices differed significantly from the point of maximum fairness of 7.5. Overall, participants demonstrated ingroup favoritism, as their scores were significantly higher than 7.5, *M* = 7.76, *SD* = 1.61, *t* (156) = 2.03, *p* = .04, *d* = 0.17. However, this effect seems to be rather small (Cohen, 1988), and overall participants did not seem to display a great amount of ingroup bias in distributing resources.

***OSIO****.* We measured how participants perceived their similarity with the ingroup and outgroup with the OSIO scale (Schubert & Otten, 2002). A positive difference score indicates that there is ingroup bias in perceived similarity. A 3 (Emotion) x 2 (Mortality salience) ANOVA on the perceived similarity difference score revealed no main effect of emotion, *F*(2, 151) = 0.63, *p* = .55, η_p_^2^ = .01, but a marginally significant main effect of mortality salience, *F*(1, 151) = 3.25, *p* = .07, η_p_^2^ = .02, in such a way that participants in the mortality salience condition showed less ingroup bias, (*M* = 0.86, *SD* = 1.26) than participants in the control condition (*M* = 1.22, *SD* = 1.26). There was no significant interaction between emotion and mortality salience, *F*(2, 151) = 2.26, *p* = .11, η_p_^2^ = .03, but simple effects analyses revealed that in the happiness condition participants who were reminded of their mortality showed significantly less ingroup bias (*M* = 0.73, SD = 1.08) than participants in the control condition (*M* = 1.62, SD = 1.30), *F*(1, 151) = 6.51, *p* = .01, η_p_^2^ = .04. In the sadness and anger conditions, there were no effects of mortality salience, both *Fs* < 1.

**S6. Correlations between facial activity (Zygomaticus) and affiliation (IOS ratings)**

*Table S1.* Correlations between Zygomaticus activity and IOS ratings (post-measurement scores) as a function of emotion (between-subjects), mortality salience (between-subjects) and social category (within-subjects).


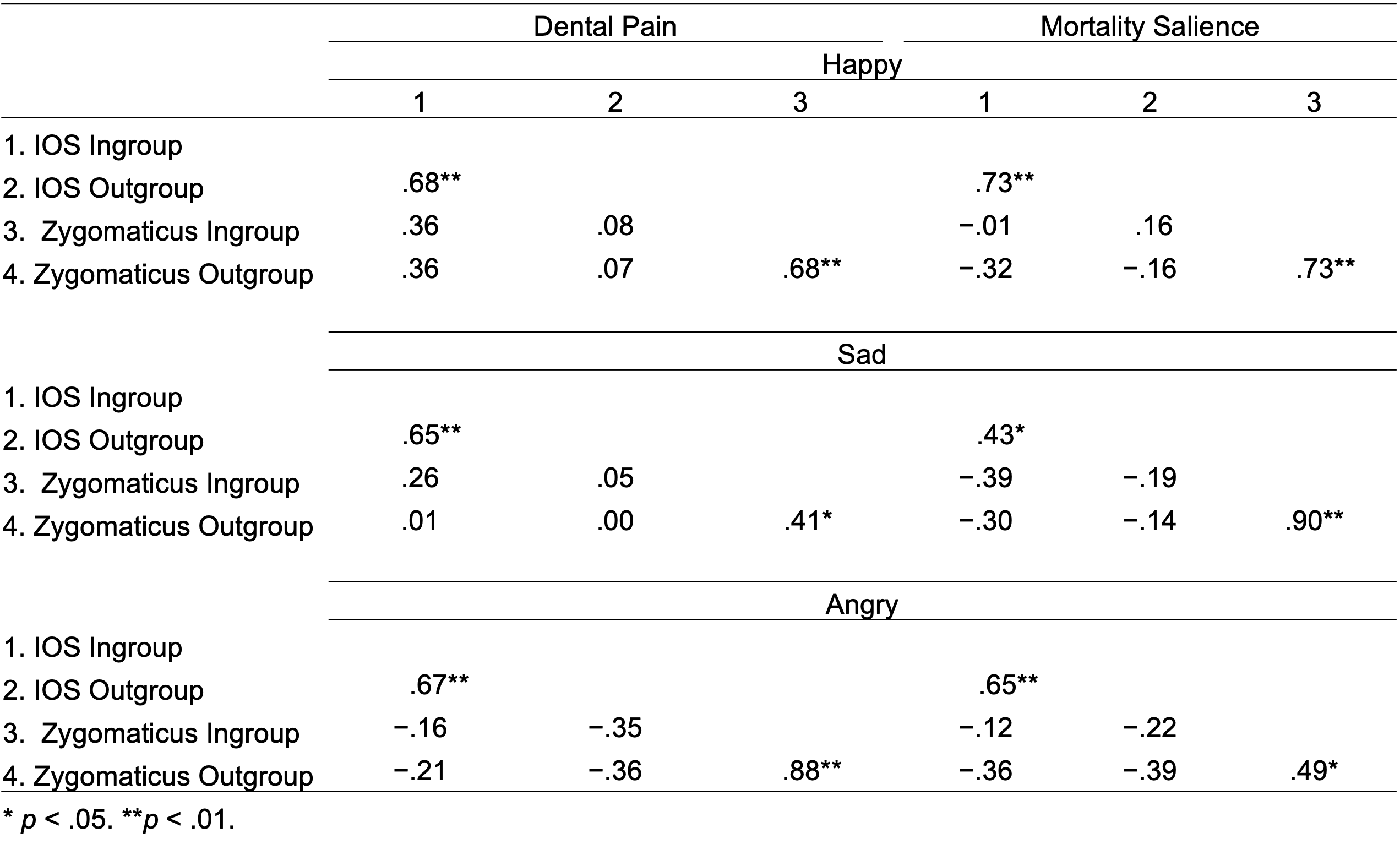


**S7. Disclosure of additional studies**

In addition to the studies reported here, we conducted an additional online study. However the design of this online study was different from the studies reported here, which meant that the results are not readily comparable. An overview of this study can be found at: https://osf.io/dd377/?view_only=f972d3983f7e4a609c87644807d947be
